# Anti-Inflammatory Effects of the Novel PIM Kinase Inhibitor KMU-470 in RAW 264.7 Cells through the TLR4-NF-κB-NLRP3 Pathway

**DOI:** 10.3390/ijms21145138

**Published:** 2020-07-20

**Authors:** Hye Suk Baek, Hyeon Ji Min, Victor Sukbong Hong, Taeg Kyu Kwon, Jong Wook Park, Jinho Lee, Shin Kim

**Affiliations:** 1 Department of Immunology, School of Medicine, Keimyung University, Daegu 42601, Korea; thortiw@naver.com (H.S.B.); mhj0221@naver.com (H.J.M.); kwontk@dsmc.or.kr (T.K.K.); j303nih@dsmc.or.kr (J.W.P.); 2 Department of Chemistry, Keimyung University, Daegu 42601, Korea; victorh@gw.kmu.ac.kr; 3 Institute of Medical Science, Keimyung University, Daegu 42601, Korea

**Keywords:** KMU-470, PIM kinase, TLR4, LPS, inflammation, NF-κB, NLRP3 inflammasome

## Abstract

PIM kinases, a small family of serine/threonine kinases, are important intermediates in the cytokine signaling pathway of inflammatory disease. In this study, we investigated whether the novel PIM kinase inhibitor KMU-470, a derivative of indolin-2-one, inhibits lipopolysaccharide (LPS)-induced inflammatory responses in RAW 264.7 cells. We demonstrated that KMU-470 suppressed the production of nitric oxide and inducible nitric oxide synthases that are induced by LPS in RAW 264.7 cells. Furthermore, KMU-470 inhibited LPS-induced up-regulation of TLR4 and MyD88, as well as the phosphorylation of IκB kinase and NF-κB in RAW 264.7 cells. Additionally, KMU-470 suppressed LPS-induced up-regulation at the transcriptional level of various pro-inflammatory cytokines such as IL-1β, TNF-α, and IL-6. Notably, KMU-470 inhibited LPS-induced up-regulation of a major component of the inflammasome complex, NLRP3, in RAW 264.7 cells. Importantly, PIM-1 siRNA transfection attenuated up-regulation of NLRP3 and pro-IL-1β in LPS-treated RAW 264.7 cells. Taken together, these findings indicate that PIM-1 plays a key role in inflammatory signaling and that KMU-470 is a potential anti-inflammatory agent.

## 1. Introduction

PIM kinases are a family of serine/threonine kinases that were named after they was identified as proviral integration sites of the Moloney murine leukemia virus [1]. The PIM kinases regulate angiogenesis, immunity, and tumor progression [2,3,4], and consist of three isoforms: PIM-1, PIM-2, and PIM-3. Recent studies demonstrated that PIM-1 plays an important role in inflammatory responses [5,6,7]. These results suggest that PIM-1 has promising potential as an anti-inflammatory target.

Inflammatory responses rely on intracellular signaling pathways and various molecular interactions. When macrophages are stimulated with lipopolysaccharides (LPS), large molecules that are a major component of the outer membrane of gram-negative bacteria, by binding to a potent immune receptor, Toll like receptor 4 (TLR4), it induces activation of nuclear factor kappa-B (NF-κB) [8] and inflammatory responses, including production and release of pro-inflammatory cytokines [9]. Recruitment of the myeloid differentiation factor (MyD88) to the plasma membrane is crucial for the intracellular inflammatory signal cascade by TLR4 [10]. This cascade results in the downstream activation of NF-κB and mitogen-activated protein kinase (MAPK), which further activates the extracellular signal-regulated kinase (ERK), c-Jun NH(2)-terminal kinase (JNK), and MAPK p38 pathways [11]. Inflammasomes, cytosolic multiprotein complexes, are responsible for caspase-1-mediated cleavage of pro-inflammatory cytokines, such as interleukin-1β (IL-1β), and consist of NLRP3, the adaptor molecule apoptosis-associated speck-like protein containing a C-terminal caspase recruitment domain (ASC) and pro-caspase-1 [9]. Moreover, activation of the NLRP3 inflammasome is regulated downstream of NF-κB [9].

In the present study, we synthesized a novel PIM kinase inhibitor, a derivative of indolin-2-one, named KMU-470, and explored its anti-inflammatory properties in LPS-treated RAW 264.7 cells. We also investigated the potential of PIM-1 as an anti-inflammatory target using PIM-1 siRNA.

## 2. Results

### 2.1. Identification of KMU-470 as A Novel PIM Kinase Inhibitor and Analysis of Inflammatory Kinase

**Figure 1:**
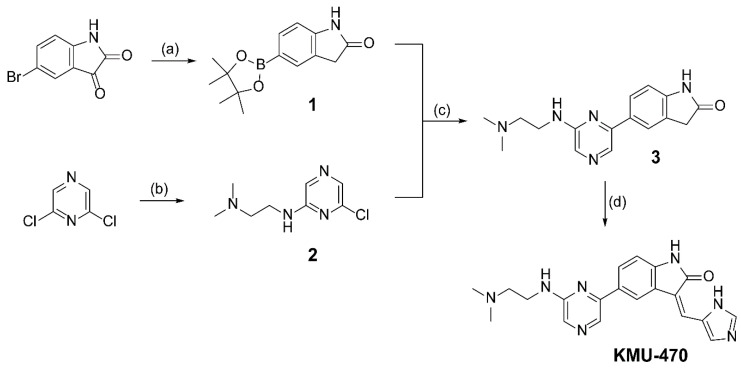
Reagents and experimental conditions: (**a**) **i**) NH_2_NH_2_, NaOH, EtOH; **ii**) bis(pinacolato)diboron, PdCl_2_(dppf), KOAc, 1,4-dioxane, microwave; (**b**) K_2_CO_3_, *N*,*N*-dimethylethylenediamine, DMF; (**c**) Pd(PPh_3_)_4_, 2M K_2_CO_3_, 1,4-dioxane/EtOH (1:1.5), microwave; (**d**) piperidine, 1*H*-imidazole-4-carbaldehyde, EtOH, microwave.

KMU-470 was synthesized from 5-bromoindoline-2,3-dione as shown in Figure 1. The compound **1** was obtained by reduction and borylation. Suzuki coupling reaction of compound **1** with pyrazine derivative, **2**, yielded compound **3**. The Knoevenagel condensation with 1*H*-imidazole-4-carbaldehyde gave KMU-470. To confirm KMU-470 as a PIM kinase inhibitor, the PIM kinase inhibitory activity of the compound was investigated. In addition, the IC_50_ values of KMU-470 were compared with those of PIM-447 and AZD-1208, known PIM kinase inhibitors. KMU-470 inhibited PIM-1, PIM-2, and PIM-3 activities with IC_50_ values of 5.6, 220, and, 6.9 nM, respectively (Table 1). However, KMU-470 showed inhibitory activities against other kinases, including inflammatory kinases, when it was tested at concentrations of 10 and 1 μM. In particular, percent activities of MAPK1, Yes, and Lyn at 1 μM concentration of KMU-470 were found to be 6, 22, and 30, respectively (Table 2).

### 2.2. Inhibition of PIM-1 Suppresses LPS-Induced Pro-IL-1β in RAW 264.7 Cells

**Figure 2:**
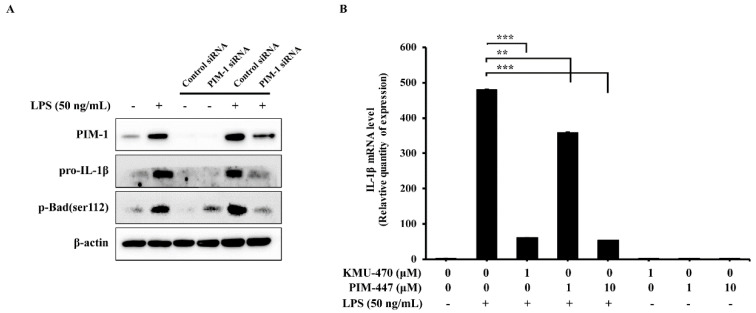
Effect of PIM inhibition on lipopolysaccharide (LPS)-mediated inflammatory response in RAW 264.7 cells. (**A**) RAW 264.7 cells transfected with negative siRNA or PIM-1 siRNA for 72 h and then treated with LPS (50 ng/mL) for 6 h. Whole cell lysates were isolated and used to measure the protein expression levels of PIM-1, pro-IL-1β, p-Bad, and β-actin by Western blot analysis. (**B**) Cells were treated with LPS (50 ng/mL) for 6 h after pre-treatment with different doses of KMU-470 (0.1, 0.5, and 1 μM) and PIM-447 (1 and 10 μM) for 1 h. Total RNA was isolated, and used to measure the mRNA expression levels of IL-1b and β-actin by RT-PCR analysis. *** *p* < 0.001, ** *p* < 0.01.

To investigate the role of PIM-1 in LPS-induced inflammatory responses in RAW 264.7 cells, we used PIM-1 siRNA and a pan-PIM kinase inhibitor. PIM-1 siRNA inhibited LPS-induced pro-IL-1β and phosphorylation of Bad, the substrate of PIM kinase (Figure 2A). Considering KMU-470 as a PIM kinase inhibitor, we next compared the anti-inflammatory potency between KMU-470 and PIM-447, a well-known pan-PIM kinase inhibitor in RAW 264.7 cells. A concentration of 1 µM KMU-470 significantly inhibited LPS-induced up-regulation of IL-1β mRNA expression in RAW 264.7 cells, whereas the same concentration of PIM-447 did not (Figure 2B).

### 2.3. KMU-470 Inhibits LPS-Induced NO Production and iNOS Expression in RAW 264.7 Cells

**Figure 3:**
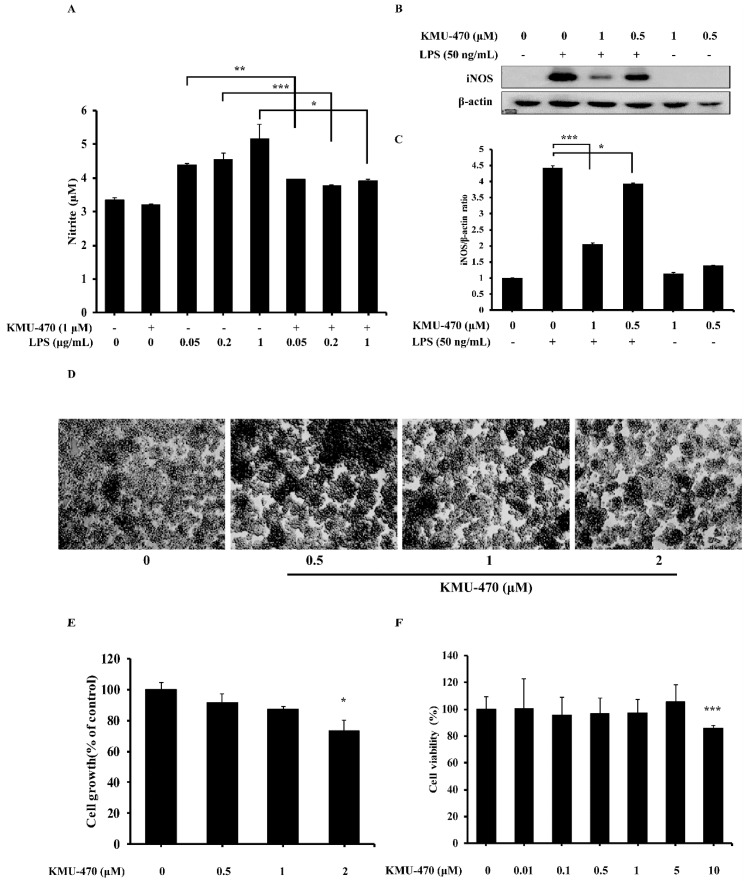
Inhibitory effect of KMU-470 on LPS-induced NO production and iNOS protein expression, and cell viability in RAW 264.7 cells. (**A**) The cells were pre-treated with KMU-470 at a concentration of 1 μM for 30 min and then treated with different concentrations of LPS for 24 h. Nitrite in the culture supernatant was collected and evaluated using Griess reagent. Absorbance was measured at 540 nm. *** *p* < 0.001, ** *p* < 0.01, * *p* < 0.05. (**B**) Cells are pre-treated with two different concentrations of KMU-470 (0.5 μM and 1 μM) for 30 min, followed by treatment with 1 μg/mL LPS for 24 h. β-actin was used as an internal control for Western blot analysis. (**C**) Image-J program was used to analyze the relative optical density of the iNOS band. (**D**) Cells were treated with various concentrations of KMU-470 for 24 h. The effects of KMU-470 on cell proliferation and morphological changes were observed by microscopy (X100). (**E**) The cell proliferation rate was determined by cell count using a trypan blue exclusion assay. *p < 0.05 (**F**) Effect of KMU-470 on cell viability was measured by XTT assay. *** *p* < 0.001.

To measure the influence of KMU-470 on NO production, we performed NO measurement in RAW 264.7 cells. KMU-470 suppressed LPS-induced NO production in RAW 264.7 cells (Figure 3A). In order to investigate the relevance of iNOS protein and the mechanism of NO suppression, the expression levels of iNOS protein were determined. KMU-470 attenuated LPS-induced up-regulation of iNOS protein in RAW 264.7 cells (Figure 3B,C). Next, we evaluated the toxicity of KMU-470 in RAW 264.7 cells. KMU-470 showed no toxicity up to 1 µM (Figure 3D–F).

### 2.4. KMU-470 Suppressed LPS-Induced TLR4 and MyD88 Expression in RAW 264.7 Cells

**Figure 4:**
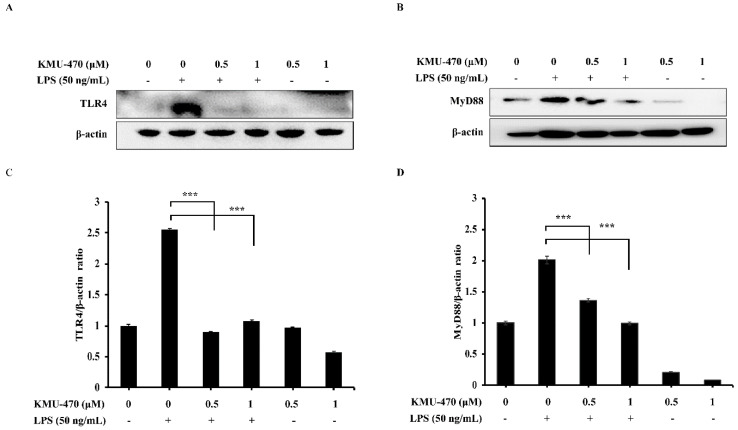
Inhibitory effect of KMU-470 on LPS-induced TLR4 and MyD88 protein expression in RAW 264.7 cells. (**A**,**B**) Cells were pre-treated with 0.5 and 1 μM of KMU-470 for 30 min prior to LPS treatment. Then, total protein was isolated after 6 h exposure to LPS. The protein expression level of TLR4 and MyD88 were assessed using Western blot analysis. (**C**,**D**) The relative optical densities of TLR4 and MyD88 bands were analyzed by the Image-J program. *** *p* < 0.001.

Next, we determined the effect of KMU-470 on the LPS-induced TLR4 and MyD88 expression in RAW 264.7 cells. KMU-470 suppressed up-regulation of TLR4 and MyD88 protein by LPS (Figure 4).

### 2.5. KMU-470 Inhibits LPS-Induced Phosphorylation of MAPKs and Activation of NF-κB in RAW 264.7 Cells

**Figure 5:**
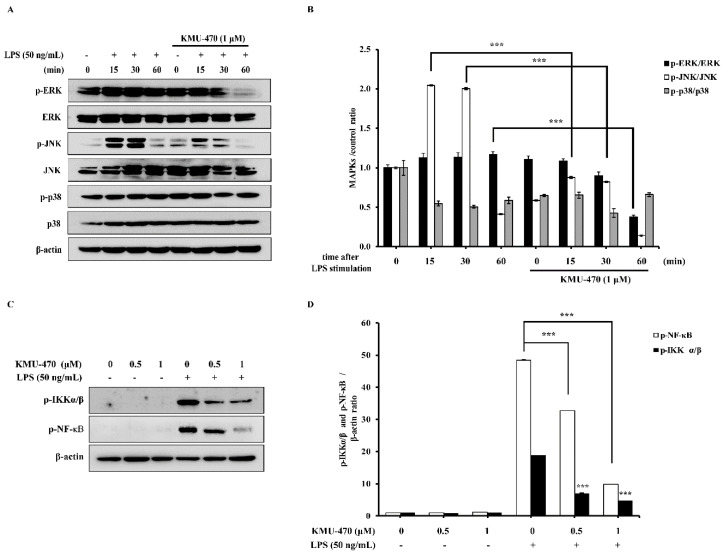
Effect of KMU-470 on LPS-induced phosphorylation of MAPKs/ IKKs/ NF-κB in RAW 264.7 cells. (**A**) Cells were pre-treated with 1μM of KMU-470 for 24 h and stimulated with LPS (50 ng/mL) at the indicated times. (**C**) Cells were pre-treated with 0.5 and 1μM of KMU-470 for 30 min prior to LPS treatment. Total proteins were isolated after 6 h exposure to LPS (50 ng/mL). The protein expression level of MAPKs, p-IKKα/β, and p-NF-κB were assessed using Western blot analyses. (**B**,**D**) The relative optical densities of MAPKs, p-IKKα/β, and p-NF-κB bands were analyzed by the Image-J program. *** *p* < 0.001.

LPS induced phosphorylation of ERK, JNK, and p38 in a time dependent manner, whereas KMU-470 significantly inhibited LPS-induced phosphorylation of JNK and ERK. However, p38 showed no change in phosphorylation level (Figure 5A,B). Additionally, LPS induced phosphorylation of IKKα/β and NF-κB, while KMU-470 significantly reduced LPS-induced phosphorylation of IKKα/β and NF-κB in RAW 264.7 cells (Figure 5C,D).

### 2.6. KMU-470 Inhibits LPS-Induced NLRP3 Inflammasome Activation and Up-Regulation of Pro-IL-1β

**Figure 6:**
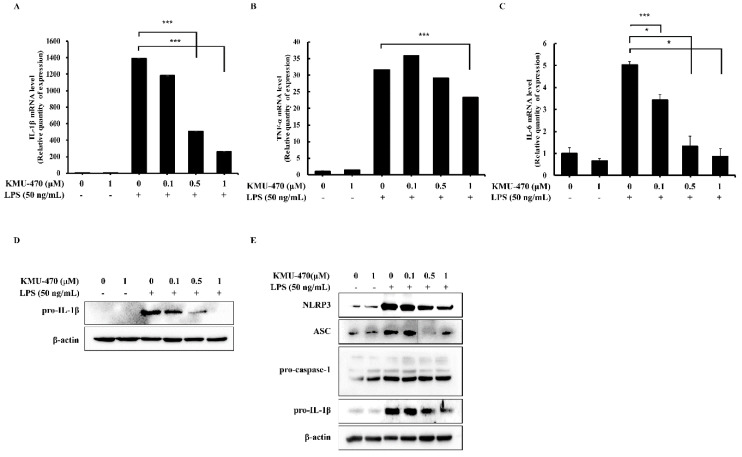
Inhibitory effect of KMU-470 on LPS-induced up-regulation of pro-inflammatory cytokines and activation of NLRP3 inflammasome in RAW 264.7 cells. (**A**–**C**) Cells were treated with KMU-470 for 30 min and stimulated with LPS 50 ng/mL for 6 h. The levels of IL-1β, TNF-α, and IL-6 were determined using real time PCR. *** *p* < 0.001, *p < 0.05. (**D**) Cells were pre-treated with 0.1, 0.5, and 1 μM of KMU-470 for 30 min and treated with LPS 50 ng/mL for 6 h, respectively. Whole cell lysates were isolated and used to measure the protein expression levels of pro-IL-1β and β-actin by Western blot analysis. (**E**) Cells were pre-treated with 0.1, 0.5, and 1 μM of KMU-470 for 30 min and treated with LPS 50 ng/mL for 6 h, respectively. Whole cell lysates were isolated and used to measure the protein expression levels of NLRP2, ASC, pro-caspase-1, pro-IL-1β, and β-actin by Western blot analysis.

We further investigated whether KMU-470 affected the NLRP3 inflammasome signal. KMU-470 showed a marked decrease in expression of NLRP3, ASC, and pro-IL-1β proteins compared with that of the LPS alone group. However, pro-caspase-1 expression was not changed by KMU-470 (Figure 6A).

### 2.7. KMU-470 Decreased LPS-Induced Pro-Inflammatory Cytokines in RAW 264.7 Cells

We investigated the effect of KMU-470 on LPS-mediated regulation of inflammatory cytokines. KMU-470 significantly decreased LPS-induced up-regulation of pro-inflammatory cytokines including IL-1β, TNF-α, and IL-6 in RAW 264.7 cells (Figure 6A–C). Moreover, KMU-470 reduced LPS-induced up-regulation of IL-1β protein (Figure 6D). These results indicate that KMU-470 effectively inhibited LPS-mediated up-regulation of pro-inflammatory cytokines.

## 3. Discussion

Members of the PIM kinase family are known as oncogenes, and many studies have been conducted on the role of PIM in tumorigenesis [12]. Recently, studies on the function of the PIM kinase family in the inflammatory response have been attracting attention. PIM-1, one of the three PIM kinases, is significantly induced by cigarette smoke in RAW 264.7 cells [7], and the inhibition of PIM-1 in a dextran sodium sulfate colitis mice model improved colitis by reducing the excessive activity of macrophages and the immune response of Th1 and Th17 [5]. In addition, PIM-1 kinase inhibitors effectively prevent the generation of cytokines, which cause allergic inflammation of the airways [6]. These findings highlight the importance of PIM-1 kinase in inflammatory responses. However, the exact mechanisms by which PIM kinases influence the inflammatory process remain unknown. In the present study, we developed a novel PIM kinase inhibitor and investigated its anti-inflammatory mechanism in RAW 264.7 cells.

Indolin-2-one is considered as a privileged scaffold for the discovery of kinase inhibitor [13]. Antitumor drugs such as Sunitinib and Intedanib are representative kinase inhibitors using an indolin-2-one scaffold [13]. Most PIM kinase inhibitors utilize the ε-amino group of Lys-67 of PIM-1 kinase as a key interaction point. For example, PIM-447 and INCB053914, under clinical trials I and II, use the pyridine ring [14,15], and AZD-1208 uses the thiazolidine-2,4-dione [16] for this purpose. Previously we reported that a series of compounds, synthesized using indoln-2-one for a scaffold and the imidazole ring for the interaction with the ε-amino group of Lys-67 of PIM-1 kinase, shows PIM kinase inhibitory activities with submicromolar IC_50_ values for all PIM-1, PIM-2, and PIM-3 kinases [17]. It was also found that the introduction of a pyrazine ring substituted with an aminoalkyl moiety, which was expected to make hydrogen bond interactions with PIM kinases, to an appropriate scaffold improved the potency of the compound against PIM kinases [18,19]. Therefore, considering the above three factors, we designed a new series of PIM kinase inhibitors that inhibits PIM kinase by competitively binding to the ATP binding pocket of PIM kinase. Indolin-2-one was used as a key scaffold, imidazole was introduced for the interaction with the ε-amino group of Lys-67 (PIM-1), and the pyrazine ring substituted with an aminoalkyl moiety was attached to the indolin-2-one ring to increase the potency of compound. We obtained potent PIM kinase inhibitors with single- or double-digit nanomolar IC_50_ values for all three PIM kinases. During screening for the anti-inflammatory activity of the PIM kinase inhibitors, KMU-470 was selected. It strongly inhibited the activities of PIM-1 and also PIM-3 kinase, with IC_50_ values of 5.6 nM and 6.9 nM, respectively (Table 1). However, this could be problematic for targeting if the inhibitor is applied to a multi-cellular system because KMU-470 is a multi-kinase inhibitor affecting the activity of several different kinases (Table 2).

First, the role of PIM-1 in the LPS-mediated inflammatory response was evaluated using PIM-1 siRNA. As shown in Figure 2A, Pim-1 siRNA inhibited LPS-induced up-regulation of pro-IL-1β and p-Bad in RAW 264.7 cells. As shown in Figure 1B, KMU-470 suppressed LPS-induced up-regulation of pro-IL-1β even at a concentration of about 1/10 that of PIM-447, a pan-PIM kinase inhibitor. This may result from KMU-470 having inhibitory activity on various inflammation-related kinases (Table 2), but it suggests that the anti-inflammatory effect of KMU-470 is superior to that of PIM-447.

iNOS is an enzyme that generates NO by converting L-arginine to L-citrulline in cells. Excess amounts of NO induce the expression of inflammatory cytokines. Consequently, the resulting inflammatory reactions can cause tissue damage, gene mutation, and nerve cell damage [20,21]. LPS promotes the production of iNOS and NO in RAW 264.7 cells, resulting in a pronounced inflammatory reaction [22,23]. A recent study has shown that LPS-induced increase of iNOS expression led to the generation of large amounts of NO, resulting in physiological dysfunction [24]. As shown in Figure 3, KMU-470 significantly decreased the generation of NO and iNOS protein expression. Accordingly, KMU-470 is considered to have anti-inflammatory effects because NO generation can be suppressed by suppressing the expression or activity of iNOS.

TLRs recognize pathogens or specific molecular components of pathogens in order to initiate the immune response [25]. Among them, TLR4 is activated by the LPS displayed on the outer membrane of Gram-negative bacteria, resulting in the activation of specific intracellular pathways, receptor dimerization, and the subsequent recruitment of a variety of adapter molecules, such as MyD88 [26]. We showed that KMU-470 inhibited LPS-induced TLR4 and MyD88 protein expression (Figure 4). LPS activates macrophages by binding to TLR4, and the LPS-initiated signaling cascade leads to activation of MAPKs and NF-κB signaling pathways [27]. It was also reported that the activation of NF-κB may occur via the MAPK signaling pathway [28,29]. These studies suggest that blocking the phosphorylation and activation of MAPKs is an important way to alleviate LPS-induced inflammation [30]. In addition, the LPS stimulus triggers activation of TLR4-MyD88, which activates NF-κB through phosphorylation and degradation of IκBα [31,32]. As shown in Figure 5, KMU-470 inhibited LPS-induced phosphorylation of IKK α/β and NF- κ B in RAW 264.7 cells.

Previous studies suggest that NF-κB is an important transcription factor for regulating the expression of iNOS and inflammatory cytokines [33]. IL-1β, TNF-α, and IL-6, are potent pro-inflammatory cytokines, and play important roles in various immune responses involving macrophages, such as inflammation [34,35,36]. Therefore, we investigated the effects of KMU-470 on pro-inflammatory cytokines in RAW 264.7 cells. KMU-470 suppressed LPS-induced up-regulation of IL-1β, TNF-α, and IL-6 at the transcriptional level (Figure 6–C). Moreover, KMU-470 inhibited LPS-induced up-regulation of IL-1β in RAW 264.7 cells (Figure 6D).

Recent studies have found that NOD-like receptors (NLRs) are important mediators in inflammatory diseases [37,38]. In particular, one of the NLRs, known as NLRP3 inflammasome, is a complex composed of NLRP3, ASC, and caspase-1, which regulates inflammatory cytokines [39]. In this study, KMU-470 inhibited LPS-induced up-regulation of NLRP3 and ASC in RAW 264.7 cells, suggesting that KMU-470 may also inhibit the inflammasome function of pro-IL-1 β cleavage.

PIM kinase inhibitors, such as AZD-1208 from Astrazeneca and PIM-447 from Novartis, have been evaluated for their potential in the pharmaceutical market of the anti-cancer field [15,40]. However, there are no reports of PIM kinase inhibitors entering the anti-inflammatory pharmaceutical market. As shown in Figure 2, it was revealed that PIM-1 kinase plays an important role in the inflammatory signaling pathway. In addition, KMU-470 was found to have inhibitory effects against multiple kinases including PIM kinase, and it was confirmed that KMU-470 has better anti-inflammatory efficacy than AZD-1208 or PIM-447, which has good PIM kinase selectivity [15,16]. These results suggest the possibility of KMU-470 entering the anti-inflammatory pharmaceutical market. Moreover, this may lead to the development of candidate compounds in the anti-inflammatory pharmaceutical market through appropriate optimizing processes such as pharmacokinetics, pharmacodynamics, in vivo study, and clinical study.

**Figure 7:**
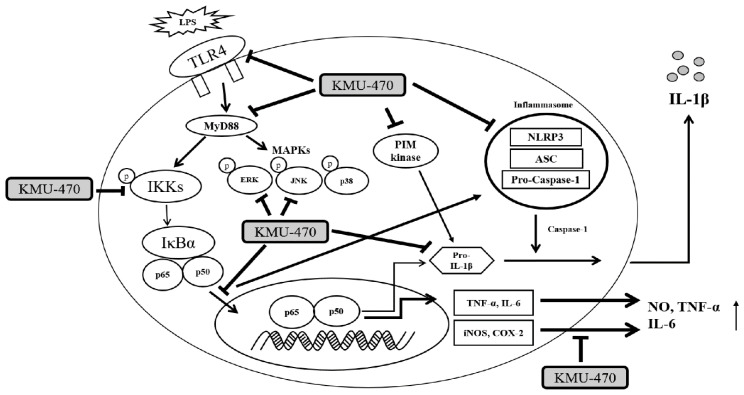
Mechanistic scheme of anti-inflammatory signaling pathways of KMU-470. LPS, lipopolysaccharide; TLR4, toll-like receptor 4; NF-κB, nuclear factor-κB; MyD88, myeloid differentiation factor 88; MAPKs, mitogen-activated protein kinases; IKK, inhibitor kappa B kinase, NO, nitric oxide; iNOS, inducible nitric oxide synthase; COX-2, cyclooxygenase-2; TNF-α, tumor necrosis factor; NRLP3, nod-like receptor family, pyrin domain containing 3; ASC, adaptor apoptosis-associated speck-like protein.

In conclusion, this study demonstrated that PIM-1 plays an important role in the inflammatory signaling associated with pro-IL-1 β up-regulation by LPS, and the novel PIM kinase inhibitor, KMU-470, has anti-inflammatory properties by regulating the TLR4/MyD88/p-IKKα/β/p-NF-κB signaling pathway and NLRP3 inflammasome (Figure 7), suggesting that this inhibitor may be developed as a potent anti-inflammatory agent.

## 4. Materials and Methods

### 4.1. Synthesis of KMU-470

#### 4.1.1. General Information

Microwave assisted reactions were performed using a CEM Discover BenchMate. Reaction completions were monitored on E. Merck silica gel F254 TLC plates. Purification of the synthesized compounds was performed by flash column chromatography using Merck Silica Gel 60 (230–400 mesh). The synthesized compounds were characterized by an ^1^H NMR on Bruker AVANCE 400 (^1^H: 400 MHz) and JEOL ECA 500 (^1^H: 500 MHz) spectrometers and their chemical shift δ values were measured in ppm with TMS as a standard reference. Mass spectra were obtained using a Waters ACQUITY UPLC, Micromass Quattro microTM API.

#### 4.1.2. 5-(4,4,5,5-Tetramethyl-1,3,2-dioxaborolan-2-yl)indolin-2-one (**1**)

A microwave vessel was filled with 5-bromoindoline-2,3-dione (0.10 g, 0.41 mmole), hydrazine hydrate (0.035 g, 1.1 mmole), and ethanol (EtOH, 1.5 mL). The mixture was irradiated for 10 min at 100 °C by applying 100 W. After addition of sodium hydroxide (0.053 g, 1.3 mmole), the mixture was irradiated for 10 min at 90 °C by applying 100 W. The mixture was acidified with 6 M hydrochloric acid (HCl). The precipitate formed by addition of cold water was obtained by filtration and washed with 6 M HCl and then with water. Drying under vacuum gave 0.07 g of 5-bromoindolin-2-one in 75% yield, which was used for the next step without further purification.

A microwave vessel was filled with of 5-bromoindolin-2-one (0.065 g, 0.31 mmole), 1,4-dioxane (2.0 mL). After the addition of bis(pinacolato)diboron (0.086 g, 0.34 mmole) and potassium acetate (KOAc) (0.091 g, 0.92 mmole), the mixture was purged with N_2_ gas for 5 min. [1,1′-Bis (diphenylphosphino) ferrocene] dichloropalladium (II) (PdCl2 (dppf)) (0.006 g, 0.009 mmole) in N_2_ gas was added to the mixture and irradiated for 10 min at 150 °C by applying 100 W. After the removal of solvent in vacuo, the residue was purified by silica gel chromatography with 1:3 ethyl acetate (EA)/dichloromethane(DCM) to give 0.02 g (25%) of the title compound. ^1^H-NMR (400 MHz, CDCl_3_): δ 9.14 (s, 1H), 7.70 (d, *J.* = 7.6 Hz, 1H), 7.67 (s, 1H), 6.91 (d, *J.* = 7.6 Hz, 1H), 3.53 (s, 2H), 1.34 (s, 12H).

#### 4.1.3. *N*-(6-Chloropyrazin-2-yl)-*N’,N’*-dimethylethane-1,2-diamine (**2**)

*N*,*N*-Dimethylethylenediamine (0.25 g, 1.7 mmol) and K_2_CO_3_ (0.46 g, 3.3 mmol) were added to 5.0 mL *N*,*N*-dimethylformamide (DMF), and the mixture was stirred for 30 min at room temperature. After addition of 2,6-dichloropyrazine (0.25 g, 2.0 mmol), the reaction mixture was stirred overnight at room temperature. The solvent was removed in vacuo. After the residue was treated with DCM, it was filtered with the aid of celite. The filtrate was collected, and the solvent was removed in vacuo. The residue was purified by silica gel chromatography with 1:9 methanol (MeOH)/DCM to give 0.27 g (75%) of the title compound. ^1^H-NMR (400 MHz, CDCl_3_): δ 7.77 (s, 1H), 7.75 (s, 1H), 5.53 (s, 1H), 3.39 (q, *J* = 5.5 Hz, 2H), 2.55 (t, *J* = 5.7 Hz, 2H), 2.26 (s, 6H).

#### 4.1.4. 5-(6-(2-Dimethylamino)ethylamino)pyrazin-2-yl)indolin-2-one (**3**)

A microwave vessel was filled with compound **1** (0.25 g, 0.097 mmole), compound **2** (0.21 g, 1.1 mmole), 1,4-dioxane (1.0 mL), EtOH (1.5 mL), and 2.0 M aqueous potassium carbonate (2.9 mL, 4.8 mmole). After the addition of tetrakis(triphenylphosphine)palladium(0) (Pd(PPh_3_)_4_) (0.033 g, 0.029 mmole) in N_2_ atmosphere, the mixture was irradiated for 10 min at 110 °C by applying 100 W. The solvent was removed in vacuo. After the residue was treated with MeOH and DCM mixture (1:9), it was filtered with the aid of celite. The filtrate was collected, and the solvent was removed in vacuo. The residue was purified by silica gel chromatography with 1:10:100 ammonium hydroxide (NH_4_OH)/MeOH/chloroform (CHCl_3_) to give 0.12 g (42%) of the title compound. ^1^H-NMR (400 MHz, CDCl_3_): δ 9.33 (s, 1H), 8.19 (s, 1H), 7.89 (s, 1H), 7.85 (d, *J* = 8.2, 1H), 7.80 (s, 1H), 6.93 (d, *J* = 8.2, 1H), 3.6 (s, 2H), 3.55-3.51 (m, 2H), 2.61 (t, *J* = 5.8 Hz, 2H), 2.30 (s, 6H).

#### 4.1.5. (*Z*)-3-((1*H*-Imidazol-5-yl)methylene)-5-(6-((2-(dimethylamino)ethyl)amino)pyrazin-2-yl)indolin-2-one

A microwave vessel was filled with compound **3** (0.12 g, 0.040 mmole), piperidine (4.0 μL, 0.04 mmole), 1*H*-imidazole-4-carbaldehyde (0.077 g, 0.81 mmole), and EtOH (2.0 mL). The mixture was irradiated for 10 min at 80 °C by applying 100 W. The solvent was removed in vacuo. The residue was purified by silica gel chromatography with 1:10:100 NH_4_OH/MeOH/CHCl_3_ to give 0.03 g (20%) of the title compound. ^1^H-NMR (500 MHz, DMSO-d_6_): δ 8.38 (s, 1H), 8.29 (s, 1H), 8.05 (s, 1H), 8.00 (s, 1H), 7.93 (d, *J* = 8 Hz, 1H), 7.88 (s, 1H), 7.66 (s, 1H), 7.99 (d, *J* = 8 Hz, 1H), 3.49 (dt, *J* = 6 Hz, *J* = 6 Hz, 2H), 2.50 (t, *J* = 6 Hz, 2H), 2.23 (s, 6H). ESI MS: m/z = 376 [M+H]^+^.

### 4.2. Reagents and Antibodies

LPS (*Escherichia coli* serotype) and PIM-447 were purchased from Sigma (St. Louis, Mo, USA) and Selleck Chemicals (Houston, TX, USA), respectively. Recombinant PIM-1, PIM-2, and PIM-3 kinases were purchased from Life Technologies (Carlsbad, CA, USA), Abcam (Cambridge, UK), and Millipore (Burlington, MA, USA), respectively. The IMAP™ FP Screening Express Kit was obtained from Molecular Devices (San Jose, CA, USA). The 5-FAM-labelled Bad peptide (5-FAM-RSRHSSYPAGT) was purchased from AnaSpec (Fremont, CA, USA). Dithiothreitol was obtained from Sigma. The primary antibodies against TLR4, p-IKK α/β, p-NFκB/P65, iNOS, MyD88, ASC, caspase-1, and NLRP3 were purchased from Cell Signaling Technology (Beverly, MA, USA). Anti-IL-1β antibody was purchased from Novus Biologicals (Centennial, CO, USA). Anti-p-Bad and anti-PIM-1 antibodies were purchased from Abcam (Cambridge, UK). Anti-β-actin antibody was purchased from Sigma-Aldrich (St. Louise, MO, USA). Anti-horse anti-mouse IgG-horseradish peroxidase (HRP) and anti-goat anti-rabbit IgG-HRP antibodies were purchased from Santa Cruz Biotechnology (Santa Cruz, CA, USA). PIM-1 siRNA was purchased from Life Technologies (Carlsbad, CA, USA).

### 4.3. Biochemical IC_50_ Value Determination

The recombinant PIM kinase, human 5-FAM-labelled Bad peptide substrate, and ATP were prepared in a reaction buffer containing 10 mM HEPES (pH 7.2), 10 mM MgCl_2_, 0.05% NaN_3_, 0.01% Triton X-100, and 2 mM DTT. Kinase reactions were performed in 384-well black flat bottom polystyrene plates (Corning 3820, Corning, NY, USA). Reagents were added to the plate as follows: 2.5 μL compound, 2.5 μL PIM kinase, 2.5 μL ATP, and 2.5 μL 5-FAM-labelled Bad peptide substrate (a final concentration of 100 nM). The final concentrations of ATP in the kinase reactions were 30 μM, 5 μM, and 20 μM for PIM1, PIM2, and PIM3, respectively. Serial dilutions of the compound (1:3) were prepared over a range from 10 μM to 0.0005 μM in reaction buffer (final reaction concentration of 1% DMSO). Kinase reactions were incubated at room temperature for 90 min, and then the reactions were stopped by the addition of 20 μL of IMAP binding reagent (solution containing 75% Buffer A: 25% Buffer B, and a 1 in 600 dilution of beads). After quenching, the plates were incubated at room temperature for 2 h, and then the fluorescence polarization measurements were carried out on an Infinity F200 (Tecan, Männedorf, Zürich, Switzerland). The percentage of inhibition was calculated for each compound concentration and the data were fitted to the equation of a 4-parameter logistic curve. IC_50_ values were obtained using GraphPad Prism (GraphPad Software, Inc., La Jolla, CA, USA).

### 4.4. Kinase Profiling

To test the specificity of the novel PIM kinase inhibitor KMU-470, a kinase profiler service was conducted by Eurofins Cerep S.A. The screening was performed with 1 and 10 μM of compound and a concentration of ATP at the Km value for each individual kinase and kinase substrate according to Eurofin’s protocols.

### 4.5. Cell Culture

RAW 264.7 murine macrophage cells were purchased from the American Type Culture Collection (ATCC, Manassas, VA, USA). RAW 264.7 cells were cultured in DMEM (GIBCO, BRL, Grand Island, NY, USA) with 10% fetal bovine serum (Welgene Inc, Daegu, Korea) and 1% antibiotics. The cells were grown at 37 °C, 5% CO_2_ in fully humidified air.

### 4.6. Cell Viability Assay

The cell viability assay was performed using a 2,3-bis (2-methoxy-4-nitro-5-sulphonyl)-2H-tetrazolium-5-carboxanilide (XTT) assay (Welgene Inc, Daegu, Korea). Briefly, RAW264.7 cells were plated at a density of 2 × 10⁴ cells/well in 96-well plates for 24 h. Cells were pre-treated with various concentrations of KMU-470 for 24 h and then incubated in 0.5 mg/mL XTT solution for 3 h at 37 °C in the dark. The optical density (OD) was measured using a microplate reader (BMG Labtech, Ortenberg, Germany) at a 450 nm wavelength. Experiments were repeated three times.

### 4.7. Measurement of NO Production

The amount of nitrite accumulated in the cell culture medium was measured directly using a Griess reagent as an indicator of NO production. RAW 264.7 cells were seeded 0.5 × 10⁶ cells/well on 12-well plates for 24 h. The cells were treated with a 1 μM concentration of KMU-470 for 30 min before LPS stimulation; they were stimulated with three different concentrations of LPS at 50, 200, and 1000 ng/mL for 24 h. Cell culture medium (100 μL) was mixed with an equal volume of Griess reagent and incubated for 10 min at room temperature. The OD was measured by spectrophotometer (BMG Labtech, Ortenberg, Germany) at 540 nm. All data are shown as the mean ± standard deviation of three experiments.

### 4.8. RNA Extraction and Real Time Quantitative PCR Analysis

Total RNA was isolated using TriZol reagent (Life Technologies; Gaithersburg, MD, USA) according to the manufacturer’s protocol. Total RNA absorbance was quantified by using Nanodrop 1000 (Thermo Fisher Scientific, Waltham, MA, USA). Complementary DNA was synthesized from total RNA using Revertra aceR qPCR RT master mix (Toyobo, Osaka, Japan). Real time quantitative PCR was performed by using the SYBR green premix (Roche Diagnostics, Mannheim, Germany) on the LightCycler 480 PCR (system Roche Diagnostics, Mannheim, Germany). The following primer sequences were used: L32 (Housekeeping gene): forward 5′-ACATTTGCCCTGAATGTGGT-3′, reverse 5′-ATCCTCTTGCCCTGATCCTT-3′; IL-1β; forward 5′-GCAACTGTTCCTGAACTCAACT-3′, reverse 5′-ATCTTTTGGGGTCCGTCAACT-3′; TNF-α; forward 5′-GACGTGGAACTGGCAGAAGAG-3′, reverse 5′-TGCCACAAGCAGGAATGAGA-3′; IL-6; forward 5′-CTGCAAGAGACTTCCATCCAG-3′, reverse 5′-AGTGGTATAGACAGGTCTGTTGG-3′.

### 4.9. Western Blot Analysis

RAW 264.7 cells were pre-treated with KMU-470 (0.5–1 μM/mL) for 24 h and stimulated with LPS (50 ng/mL) for 6 h. After stimulation, total cells were harvested, washed with PBS buffer, and then the total protein was extracted with a RIPA buffer (0.1 mM sodium orthovanadate, 137 mM NaCl, 15 mM EGTA, 0.1% Triton X-100, 25 mM MOPS, 15 mM MgCl_2_, 100 μM phenylmethylsulfonyl fluoride, and 20 μM leupeptin, adjusted to pH 7.2). The cell culture media supernatants were precipitated using TCA (trichloroacetic acid, protocol by Luis Sanchez 2001). A BCA protein assay kit (Thermo Scientific, Wilmington, USA) was used for the determination of the protein concentration. Cell lysates of equal protein concentrations were prepared in 5× sample buffer (ELPIS Bio Technology, Daejeon, Korea). Lysates were separated by 10% SDS-PAGE gel electrophoresis and then transferred onto Immobilon-P membranes (Millipore Corporation, Bedford, MA, USA). The membranes were rinsed and incubated with blocking buffer (5% non-fat milk in 1× TBS/0.1% Tween^®^20) for 1 h. Then, the membranes were incubated with primary antibodies overnight at 4 °C and then with horseradish peroxidase-conjugated secondary antibodies for 1 h. Immunoreactive proteins were detected using the ELC Western blot detection system kit (Millipore). Signal intensity was measured using the Chemi Image documentation system (Fusion Fx7, VILBER LOUTMAT). Protein expression levels were quantified using the Image J program.

### 4.10. Small Interfering RNA Transfection

The RAW 264.7 cells were seeded in six-well plates and transfected with PIM-1 siRNA (s10527, Life Technologies) or control siRNA using a transfection reagent (Santa Cruz biotechnology). All transfections were carried out with Lipofectamine RNAiMAX (Life Technologies). RAW 264.7 cells were transfected with 30–45 nM siRNA per well. After 72 h, cells were used for further experiments.

### 4.11. Statistical Analysis

All data are presented as the mean and standard error of mean (SEM) and experiments were repeated three times. Data analysis was performed using SPSS 25.0 software program. A *p*-value < 0.05 was considered significant.

## Figures and Tables

**Table 1:** PIM kinase inhibitory activities of KMU-470.

Compound	Inhibitory Activity IC_50_ (nM) *		
	PIM-1	PIM-2	PIM-3
KMU-470	5.6	220	6.9
AZD-1208	3.0	6.0	3.0
PIM-447	2.1	12	0.9

* Average of two independent experiments.

**Table 2:** Kinase inhibitory activities of KMU-470 against inflammation-related kinases.

KMU-470 10 (μM)		KMU-470 1 (μM)	
Kinase	Activity (% Control)	Kinase	Activity (% Control)
MAPK1 (h)	−18	MAPK1 (h)	6
Yes (h)	−3	Yes (h)	22
Blk (h)	5	Blk (h)	53
Fgr (h)	8	Fgr (h)	42
Lyn (h)	10	Lyn (h)	30
Lck (h)	14	Lck (h)	41
TYK2 (h)	14	TYK2 (h)	54
JAK3 (h)	15	JAK3 (h)	38
Fyn (h)	16	Fyn (h)	97
JAK1 (h)	20	JAK1 (h)	61
Itk (h)	21	Itk (h)	75
Syk (h)	21	Syk (h)	82
JAK2 (h)	23	JAK2 (h)	107
JNK1α1 (h)	34		
Hck (h)	37		
Pyk2 (h)	41		
Csrc (h)	43		
Bmx (h)	47		
Txk (h)	50		
Tec (h) activated	71		
SAPK2a (h)	77		
BTK (h)	80		
ZAP-70 (h)	100		

## Abbreviations

LPS	lipopolysaccharide
TLR4	toll-like receptor 4
NF-κB	nuclear factor-κB
MyD88	myeloid differentiation factor 88
MAPKs	mitogen-activated protein kinases
IKK	inhibitor kappa B kinase
NO	nitric oxide
iNOS	inducible nitric oxide synthase
COX-2	cyclooxygenase-2
TNFα	tumor necrosis factor alpha
IL-1β	interleukin-1β
IL-6	interleukin-6
NRLP3	nod-like receptor family pyrin domain containing 3
ASC	adaptor apoptosis-associated speck-like protein
OD	optical density
